# The Time Course of Quadriceps Strength Recovery After Total Knee Arthroplasty Is Influenced by Body Mass Index, Sex, and Age of Patients: Systematic Review and Meta-Analysis

**DOI:** 10.3389/fmed.2022.865412

**Published:** 2022-05-25

**Authors:** Armin H. Paravlic, Cécil J. Meulenberg, Kristina Drole

**Affiliations:** ^1^Institute for Kinesiology Research, Scientific Research Center Koper, Koper, Slovenia; ^2^Faculty of Sport, Institute of Kinesiology, University of Ljubljana, Ljubljana, Slovenia; ^3^Faculty of Sport Studies, Masaryk University, Brno, Czechia

**Keywords:** knee osteoarthritis, functional performance, rehabilitation, voluntary activation, body mass index (BMI), total knee replacement

## Abstract

**Introduction:**

For patients with osteoarthritis who have undergone total knee arthroplasty (TKA), quadriceps strength is a major determinant of general physical function regardless of the parameters adopted for functional assessment. Understanding the time course of quadriceps strength recovery and effectiveness of different rehabilitation protocols is a must. Therefore, the aim of this study was to: (i) determine the magnitude of maximal voluntary strength (MVS) loss and the time course of recovery of the quadriceps muscle following TKA, (ii) identify potential moderators of strength outcomes, and (iii) investigate whether different rehabilitation practices can moderate the strength outcomes following TKA, respectively.

**Design:**

General scientific databases and relevant journals in the field of orthopedics were searched, identifying prospective studies that investigated quadriceps’ MVS pre-to post-surgery.

**Results:**

Seventeen studies with a total of 832 patients (39% males) were included. Results showed that in the early post-operative days, the involved quadriceps’ MVS markedly declined, after which it slowly recovered over time in a linear fashion. Thus, the greatest decline of the MVS was observed 3 days after TKA. When compared to pre-operative values, the MVS was still significantly lower 3 months after TKA and did not fully recover up to 6 months following TKA. Furthermore, a meta-regression analysis identified that the variables, time point of evaluation, patient age, sex, and BMI, significantly moderate the MVS of the quadriceps muscle.

**Conclusion:**

The analyzed literature data showed that the decrease in strength of the involved quadriceps muscles following TKA is considerable and lasts for several months post-surgery. Therefore, we recommend to specifically target the strengthening of knee extensor muscles, preserve motor control, and apply appropriate nutrition to ensure a holistic quadriceps muscle recovery. Since age, sex, and BMI were found to be moderating factors in patients’ recovery, further research should include specific analyses considering these moderators.

## Introduction

Osteoarthritis (OA) is the most common cause of disability among older adults (1) due to marked decline in muscle strength and flexibility, combined with the presence of knee joint pain (2). When more conservative treatments fail (3–5), total knee arthroplasty (TKA) as a surgical replacement of a degenerated or malformed joint is prescribed to patients with OA (6, 7). Since both the incidence and prevalence of OA increase with age (8, 9), longer life expectancy that is being faced globally will result in an increase in primary TKA rates, which are anticipated to increase by 673% until 2030 (10).

Considering the hospital stay duration of 3–4 days (11) and muscle disuse in patients with OA (12, 13), along with surgical procedure, the whole TKA has the potential to elicit a considerable decline in functional performance of hospitalized individuals (14, 15).

For patients with OA who have undergone TKA, quadriceps strength is a major determinant of general physical function regardless of the parameters adopted for functional assessment (2, 16, 17). Weakness of the quadriceps muscle persists for a long period of time (18) and may not achieve the pre-operative levels (19) of the involved leg up to 6 months post-surgery (20, 21). Furthermore, when compared to the uninvolved or healthier limb or even healthy counterparts, quadriceps weakness can last even longer (22, 23). Therefore, it is very important to determine the time course of quadriceps strength loss and its recovery, i.e., comparing its pre-to-post-surgery values, in order to attenuate detrimental loss and plan an adequate rehabilitation process.

Previous literature reviews that aimed to investigate the post-surgery time course of recovery have compared the lower limb strength of patients with TKA with healthy age-matched control subjects (19), which does not completely visualize the true recovery pathway because it does not take into consideration the pre- to post-surgery strength levels of individual subjects. A study of Moon et al. (24) only included isokinetic strength assessment (24) and reported the exclusion of three studies because of isometric strength assessment. However, experiments have shown that various strength evaluation modalities, including isometric and isokinetic models are comparably valid and reliable tools for quadriceps muscle strength assessment in patients with TKA (25), and that discrepancies of muscle weakness estimates could be observed (18). Moreover, there is evidence that the contralateral leg (i.e., pre-operatively apparently healthy leg) experiences a significant decline in MVS and other functional outcomes (26, 27); thus, it might not represent a valid comparator, especially in the long term when it becomes more painful than the involved leg (27). Given that the previous qualitative reviews and original investigations were limited by the above-mentioned issues, there is a need to summarize and critically analyze the vast amount of data that evaluate the maximal voluntary isometric (MViC) strength of quadriceps muscles in both the pre- and post-surgery periods using involved leg as its own comparator. Since a positive correlation has been found between higher BMI and development of degenerative knee disease, it has been proposed that BMI could affect the outcomes after TKA (28). It is known that the weight of patients with TKA fluctuates over time, and there is evidence that body mass index (BMI) has a negative influence on functional outcomes (26, 29). However, some studies found no important differences (30, 31); thus, the influence of BMI on clinical and functional outcomes following TKA is still inconclusive. Thus, there is a need to control its effect on strength outcomes given that strength is a body size-dependent measure (32). Additionally, the aforementioned studies have not evaluated the potential moderators of the observed effects, which would certainly contribute to existing knowledge of the recovery of Patients who underwent TKA and, thus, improve the post-rehabilitation processes.

Several studies report sex differences in both incidences and outcomes after musculoskeletal injuries. Higher incidence of anterior cruciate ligament injuries, as well as higher prevalence of OA, has been reported in females compared to males (33); however, there is still an abundance of research that does not conduct a sex-specific analysis. Furthermore, research shows that being female (9), older, and being obese (34) also predispose an individual to have worse symptoms and greater disability after OA. Furthermore, having more severe OA pre-TKA often results in worse post-operative outcomes (35). Research showed that women in general have worse outcomes after TKA (36), while Singh et al. (37) found that women also have a greater risk of experiencing moderate to severe pain 2 and 5 years post-TKA. Even though lower levels of estrogen, which occur in post-menopausal women, have been associated with reduced cartilage health (34), estrogen is responsible for boosting the immune system by increasing antibody production; however, testosterone does the opposite (38) by decreasing antibody production. Thus, males are shown to have a higher risk of developing post-operative infections. Another study showed that being female, older age, and having deficits in quadriceps strength are related to altered gait mechanics post-TKA (39). However, some studies found that women tend to achieve greater improvements on different knee function evaluation scales (Oxford knee score, etc.), and that men report a higher final level of function (40).

As mentioned above, another possible moderator in recovery after TKA is patients’ age. Although it was thought that older patients are at risk of having worse outcomes, a recent study showed that younger patients have suboptimal recovery after TKA (41).

Because of inconclusive research results, it is still unknown how different patient characteristics influence rehabilitation outcomes after TKA. Therefore, the aims of this meta-analysis are: (i) to determine the magnitude of maximal voluntary strength (MVS) loss and recovery of the quadriceps muscles in periods after TKA by (a) comparing the pre to post measurements of strength within-subjects and by (b) comparing patients with TKA with healthy age-matched controls; (ii) to investigate whether different rehabilitation practices can moderate strength outcomes following TKA; (iii) to evaluate whether sex, age, and BMI of the patients might influence strength loss and/or recovery following TKA. Therefore, we hypothesized that: (i) the involved quadriceps MVS will recover in a linear fashion and will be time-dependent; (ii) within-subjects analysis will show that MVS recovers earlier than when it was compared to healthy age-matched controls; (iii) patients’ age, sex, and BMI will moderate MVS outcomes following TKA.

## Methods

### Search Strategy

Both the systematic review and the meta-analysis were conducted in accordance with the Preferred Reporting Items for Systematic Reviews and Meta-Analyses (PRISMA) statement guidelines (42). Thus, a systematic search of the research literature published in peer-reviewed journals was conducted for experimental trials studying the time course of the recovery of quadriceps muscle function following TKA surgical procedure among adult subjects. To carry out this review, English language literature searches of the MEDLINE/PubMed, Google Scholar, Science Direct, PEDro, SAGE Journal, CINAHL, SPORTDiscus, Embase, and Cochrane databases were conducted from June 2021 to August 2021. Electronic databases were searched as well using the following keywords and their combinations: “total knee arthroplasty,” “knee replacement surgery,” “functional performance,” “functional impairment,” “quadriceps,” “knee extensors muscles,” “muscle strength,” “torque,” “force,” “MVC,” “rehabilitation,” “body mass index,” and “BMI.” Reference lists of each included article were also scanned to additionally identify relevant studies. When the database search was conducted, it was free of any restrictions such as language of the original publication or year in which the article was published.

### Study Selection

Eligible studies were selected by (a) *Population*: studies recruiting both male and female adult participants in any age category who were scheduled to undergo primary unilateral TKA for the treatment of knee osteoarthritis; (b) *Comparison*: the maximal voluntary muscle strength (MVS) was compared with pre-surgery values of the involved leg across different time points during the follow-up period after surgery (i.e., following 3 days after, 14 days, and 1 month, 1.5–3 months, 6 months, 12 months, 24 months, and 60 months after); Patients vs. healthy aged-control subjects; pre-surgery treatment vs. post-surgery usual care (UC); UC vs. outpatient professionally guided rehabilitation (OPGR) treatment; (c) *Outcome:* the MVS normalized by kilograms of body mass or body mass index (BMI) (MViC-norm). We chose to include only the normalized values of strength given that MVS is body size-dependent (32).

Studies were excluded according to the following criteria: (a) studies that included isokinetic measurements of MVS, (b) studies that reported non-normalized values of MVS, (c) studies that did not evaluate the patients before surgery, (d) studies that included patients scheduled for revision and/or bilateral TKA, and (e) studies from which not enough information could be extracted to calculate the effect sizes.

### Screening Strategy

**FIGURE 1:**
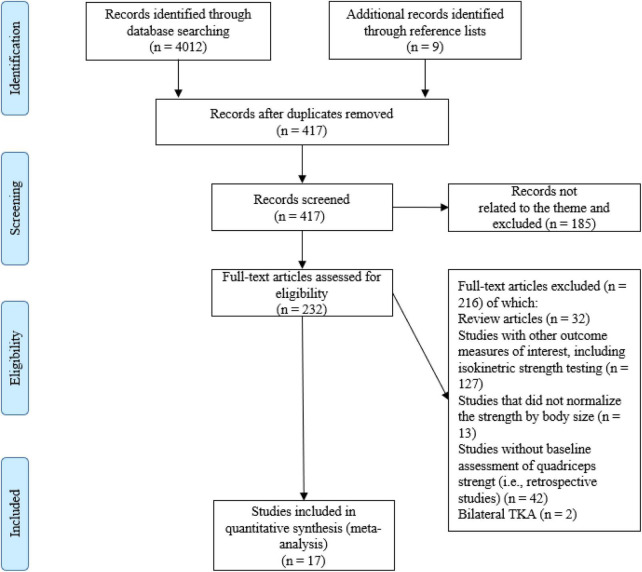
Flow diagram of the process of study selection.

Initially, one researcher (AP) performed the literature search, along with study identification, screening, quality assessment, and data extraction. Subsequently, another researcher (CJWM) checked all the data independently. First, the titles were initially screened by the reviewers during the electronic searches to assess the suitability of the articles, and all articles beyond the scope of this review were excluded. Second, the abstracts were assessed using the predetermined inclusion and exclusion criteria. Third, the full texts of the remaining articles that met the inclusion criteria were retrieved and included in the ongoing procedure and reviewed by the two reviewers to reach a final decision on inclusion in the meta-analysis. Finally, the reference lists from the retrieved manuscripts were also examined for any other potentially eligible articles. Any disagreements between the reviewers were resolved by consensus or arbitration by a third reviewer (KD). If the full text of any article was not available, the corresponding author was contacted by mail or ResearchGate. The process of study selection, as described above, is illustrated in Figure 1.

### Data Extraction

The Cochrane Consumers and Communication Review Group’s data extraction protocol was used to extract participant information, including sex, age, sample size, training status, description of the intervention, study design, and study outcomes (43). The extraction was conducted by one author (AP), while a second author (CJWM) checked the extracted data for accuracy and completeness. Disagreements were resolved by consensus or by a third reviewer (KD). The reviewers were not blinded to authors, institutions, or manuscript journals. In these studies, where data were shown in figures or graphs, either the corresponding author was contacted to get the numerical data to enable analysis or the Web Plot Digitizer software (Version 3.10, Austin, TX, United States) was used to extract the necessary data.

### Quality Assessment

Quality assessment was conducted by one author (AP), while another (CJWM) checked all the data independently. Disagreements were resolved by consensus. For observational or non-randomized surgical studies, the 12-item Methodological Index for Non-randomized Studies (MINORS) was used (44). MINORS is a valid instrument and is designed to assess the methodological quality of non-randomized surgical studies, whether comparative or non-comparative. Each item was scored a “0” (not reported), “1” (not adequately reported), or “2” (adequately reported). The maximum score was 24 for comparative studies.

The quality of evidence was assessed using the Grading of Recommendations Assessment, Development and Evaluation system, and classifications were made as follows: high quality, moderate quality, low quality, and very low quality (45). However, several reasons might lead to degradation of the quality of evidence. Thus, in this study, we considered the following criteria when assessing confidence in evidence: design limitation (if the majority of studies in the meta-analysis had a MINORS score < 14), imprecision based on small sample size (<300 for each pooled outcome), and inconsistency of the results (substantial heterogeneity within effect estimates, *I*^2^≥ 50%). This review did not consider the indirectness criterion because the eligibility criteria ensured a specific population with relevant outcomes.

### Statistical Analysis

The collected data for this review are presented as mean ± standard deviation (SD). The meta-analyses were performed using the Comprehensive Meta-analysis software (Version 2.0, Biostat Inc., Englewood, NJ, United States). Effect size is calculated according to the following formula: $E\,S=\frac{M_{p\,o\,s\,t}-M_{p\,r\,e}}{S\,D_{p\,o\,o\,l\,e\,d}}$. In accordance with Hedges (46), this formula was adjusted for sample size: $\text{J}=1-\left(\frac{3}{4\,N\,i-1}\right)$, where Ni is the total sample size of the intervention group minus one. Mean differences and 95% confidence intervals (*CI*s) were calculated for the included studies. The *I*^2^ measure of inconsistency was used to examine between-study variability; values of 25, 50, and 75% represent low, moderate, and high statistical heterogeneity, respectively (47). A fixed effect model of meta-analysis was applied in all comparisons to determine the pooled effect of TKA on MVS, whereas a random-effects model of meta-analysis was used only when statistically high heterogeneity was detected. In addition, a sensitivity analysis was conducted using both fixed and random effects for major comparisons. If the effect and *CI*s of the sensitivity analysis were similar between the two models, the results were considered robust.

Additionally, a meta-regression based on method of moments was performed to examine whether the time points of evaluation after surgery, as well as the participants’ age, sex ratio, and BMI, as continuous variables, may predict alterations in MVS following TKA. Also, we performed an additional analysis by excluding studies with lower methodological quality (48, 49). Moreover, a sub-analysis was performed to investigate the magnitude of the effect regarding rehabilitation treatment (i.e., pre-operative vs. usual care vs. OPGR) and the patients’ BMI (<30 or ≥30 kg/m^2^).

The chance of the true effect being trivial, beneficial, or harmful was interpreted using the following scale: 25–75% (possibly), 75–95% (likely), 95–99.5% (very likely), and 99.5% (most likely), according to a previous approach developed by Hopkins (50). Publication bias was assessed by examining the asymmetry of the funnel plots by Egger’s test, and significant publication bias was considered if *p* < 0.1. The magnitude of TKA effects on muscle strength was interpreted as changes using the following criteria: trivial (<0.2), small (0.21–0.6), moderate (0.61–1.2), large (1.21–2), very large (2.01–4), and extremely large (>4) (50). A significance level of *p* ≤ 0.05 was used for all the analyses.

## Results

### Study Selection

**FIGURE 2:**
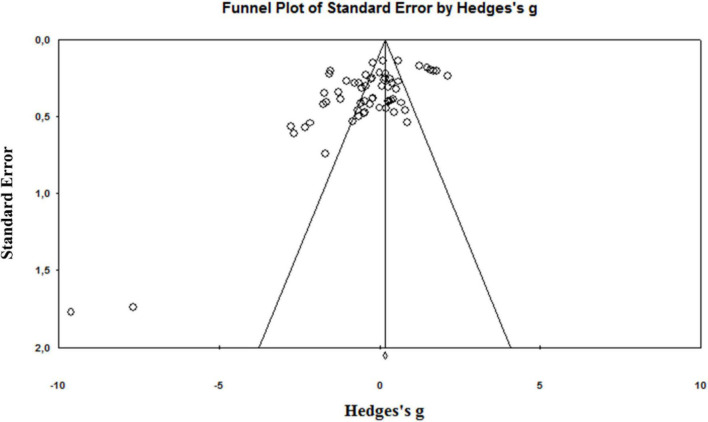
Funnel plot of the standard differences in means vs. standard errors for all included effect sizes.

A total of 4,012 articles were identified by the literature search (Figure 1). Following the removal of duplicates and the elimination of articles based on title and abstract screening, 417 studies remained. An evaluation of the remaining 417 studies was conducted independently by two researchers. When studies presented different subgroups [e.g., receiving different pre-and/or post-operative physical therapy regimens (15, 20, 51–59), had patients with or without diabetes (60), or examined diverse approaches of surgery (57, 61–64)], these were considered to be different pre-post comparisons, which led to 65 independent comparisons. Following the final screening process, 17 studies were included in the systematic review and meta-analysis. Egger’s test was performed to provide statistical evidence of funnel plot asymmetry (Figure 2), and the results indicated publication bias for all the analyses (*p* < 0.1).

### Study Characteristics

After the literature search, 16 eligible articles were found and included with their details regarding sample size, measures, results, and additional comments (see Supplementary Table 1). All the studies included reported measurements of MViC, although different measures to normalize the data, such as kg of body mass (20, 49, 51, 52, 57, 60, 61, 65–67) or BMI (7, 17, 22, 48, 68, 69), were adopted. Furthermore, only two studies used an untreated healthy aged-matched group for comparison (22, 66). All but four studies (48, 65, 69) had more than one measurement assessment following operation, ranging from 3 days to 60 months.

In all the included studies, first unilateral TKA was performed primarily because of OA. All but five studies did not report the surgical approach used (49, 52, 56, 60, 61). The standard medial parapatellar approach was mostly used (7, 20, 48, 51, 57, 59, 64–67, 70)

In large majority of the studies, the usual care of post-operative rehabilitation physical therapy was assessed. However, in one study, only a pre-surgery exercise program was implemented (56). Six studies carried out a post-operative treatment of professionally guided progressive rehabilitation (7, 17, 51, 52, 56, 68). One study performed neuromuscular electrical stimulation (NmES) in addition to outpatient professionally guided rehabilitation (OPGR) (20).

### Subject Characteristics

The pooled sample size of the 17 studies yielded 832 participants, where the typical sample size of the individual studies ranged from 8 to 119 subjects per group. Sex information was available in all, with the exception of one study (69), resulting in 39% male subjects for this meta-analysis. The age of the patients was stated in all the studies, with a pre-surgery age ranging from 60.6 to 75.9 years (mean: 67.3 years). BMI was reported in almost all of the studies, with a range of 24.6–35 kg/m^2^ (mean: 29.5 kg/m^2^).

### Quality Assessment

The mean MINORS score for the 17 included observational studies was 18.8 ± 3 (Table 1). All the investigated studies received a maximum of 2 points for the following items: clearly stated aim, inclusion of consecutive patients, prospective collection of data, and adequate statistical analyses of data. In addition, nine studies did not report the bias of assessment of the study endpoint; two studies had more than 5% loss of subjects during the follow-up period; seven studies did not have a prospective calculation of the study size; four studies did not include comparison groups; four studies reported baseline in-equivalence. Furthermore, the quality of evidence assessment is presented in Table 2.

**TABLE 1:** Quality assessment of the included studies (*n* = 17).

Study name	Item 1	Item 2	Item 3	Item 4	Item 5	Item 6	Item 7	Item 8	Item 9	Item 10	Item 11	Item 12	Total
Bade and Stevens-Lapsley (52)	2	2	2	2	0	2	0	2	2	0	1	2	17
Collados-Maestre et al. (61)	2	2	2	2	2	2	2	2	2	2	2	2	24
Holm et al. (65)	2	2	2	1	1	1	2	2	0	0	0	2	15
McKay et al. (56)	2	2	2	1	0	1	1	1	2	2	2	2	18
Mizner et al. (48)	2	2	2	2	0	2	2	0	0	0	0	2	14
Mizner et al. (17)	2	2	2	2	2	2	2	2	0	0	0	2	18
Mizner et al. (7)	2	2	2	2	0	2	2	0	1	2	1	2	18
Mizner et al. (68)	2	2	2	2	0	2	2	0	1	2	1	2	18
Nutton et al. (57)	2	2	2	2	2	2	1	0	2	2	2	2	21
Pua et al. (49)	2	2	2	2	0	2	1	0	0	0	0	2	13
Smith et al. (22)	2	2	2	2	0	1	2	0	2	2	2	2	19
Stevens et al. (69)	2	2	2	1	1	1	2	0	1	2	2	2	18
Stevens-Lapsley et al. (66)	2	2	2	2	0	2	2	2	2	2	1	2	21
Stevens-Lapsley et al. (20)	2	2	2	2	1	2	1	2	2	2	1	2	21
Wada et al. (60)	2	2	2	2	0	2	2	2	2	2	2	2	22
Yoshida et al. (51)	2	2	2	1	2	1	2	2	2	2	2	2	22
Paravlic et al. (67)	2	2	2	2	1	2	0	2	2	2	2	2	21

*Item 1, a clearly stated aim; Item 2, inclusion of consecutive patients; Item 3, prospective collection of data; Item 4, endpoints appropriate to the aim of the study; Item 5, unbiased assessment of the study endpoint; Item 6, follow-up period appropriate to the aim of the study; Item 7, loss in follow-up less than 5%; Item 8, prospective calculation of study size; Item 9, an adequate control group; Item 10, contemporary groups; Item 11, baseline equivalence of the groups; Item 12, adequate statistical analyses.*

**TABLE 2:** Quality of the evidence assessment using the Grading of Recommendations Assessment, Development, and Evaluation (GRADE) system.

Outcome (MVS)	Trials (*n*)	Participants (*n*)	Effect size	LLCI	HLCI	*I* ^2^	MINORS score	Quality of evidence (GRADE)
3 days after	1	24	–2.65	–3.85	–1.46	0	15	Moderate quality*
14 days after	2	107	–1.77	–2.13	–1.41	0	18	Moderate quality*
1 month after	4	130	–1.47	–2.01	–0.94	81	17.5	Low quality*^,©^
1.5–3 months after	7	282	–0.38	–0.60	–0.17	52	19	Low quality*^,©^
6 months after	7	449	0.28	–0.02	0.59	79	21	Moderate quality^©^
12 months after	6	496	0.77	0.30	1.24	85	20	Moderate quality^©^
24 months after	1	237	1.64	1.37	1.92	0	24	Moderate quality*
60 months after	1	237	1.68	1.40	1.96	0	24	Moderate quality*

*Abbreviations: MVS, maximum voluntary strength; n, number; GRADE, Grades of Recommendation, Assessment, Development, and Evaluation; LLCI, lower limit of 95% confidence interval; HLCI, higher limit of 95% confidence interval.*

*Downgraded because of imprecision based on small sample size (<300 for each pooled outcome).

*^©^Downgraded because of inconsistency of results (substantial heterogeneity within effect estimates, I^2^≥ 50%).*

## Overall Findings

### Maximal Voluntary Isometric Contraction Strength (Normalized) Recovery Following Total Knee Arthroplasty Surgery

#### Maximal Voluntary Isometric Contraction-Norm Recovery Following Total Knee Arthroplasty Surgery for Time Points Less Than 1.5 Months

**FIGURE 3:**
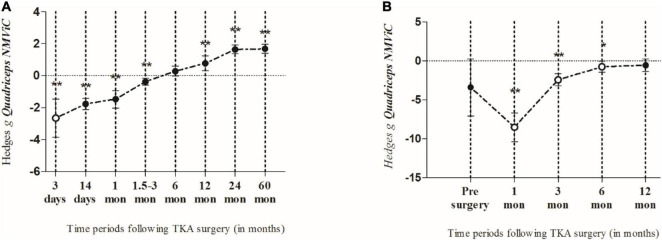
The summarized effects of more than one effect size (closed circles) and one effect size only (open circles), demonstrating the time course of quadriceps muscle maximal voluntary isometric strength normalized by body weight (NMViC) **(A)** comparison of pre to post within patients and **(B)** compared with healthy age-matched controls. Data are presented as effect sizes and lower and upper limits of 95% confidence interval.

One study only assessed the MVS 3 days post-surgery and showed a most likely very large harmful effect on MVS (*ES* = −2.65; 95% *CI* −3.85 to −1.46; *n* = 1; *I*^2^ = 0%) (Table 3 and Figure 3A). The evidence was downgraded from high quality to moderate quality because of imprecision (sample size < 300) (Table 2).

**TABLE 3:** Effects of total knee arthroplasty on maximal isometric strength normalized to kilograms of body mass or BMI, considering different time periods of post-surgery evaluation.

Independent variables	*ES*	*SE*	95% *CI*	*Z*	*p*	*n*	*I*^2^ [%] (*p*)
**3 days after TKA**							
Fixed effect	–2.65	0.61	-3.85 to -1.46	–4.35	< 0.001	1	0.0 (1.0)
**14 days after TKA**							
Fixed effect	–1.77	0.18	-2.13 to -1.41	–9.58	< 0.001		
Random effect	–1.77	0.18	-2.13 to -1.41	–9.58	< 0.001	4	0.0 (0.425)
**24–30 days after TKA**							
Fixed effect	–1.22	0.11	-1.42 to -1.01	–11.50	< 0.001		
Random effect	–1.47	0.27	-2.01 to -0.94	–5.43	< 0.001	14	81.09 (<0.001)
**One and a half month to 3 months after TKA**							
Fixed effect	–0.34	0.07	-0.48 to -0.20	–4.86	< 0.001		
Random effect	–0.38	0.11	-0.60 to -0.17	–3.51	< 0.001	20	51.8 (0.004)
**6 months after TKA**							
Fixed effect	0.37	0.06	0.25 to 0.50	5.78	< 0.001		
Random effect	0.28	0.15	-0.02 to 0.59	1.86	0.063	14	79.02 (<0.001)
**12 months after TKA**							
Fixed effect	0.90	0.09	0.72 to 1.07	10.11	< 0.001		
Random effect	0.77	0.24	0.30 to 1.24	3.23	0.001	10	84.81 (<0.001)
**24 months after TKA**							
Fixed effect	1.64	0.14	1.37 to 1.92	11.70	< 0.001		
Random effect	1.64	0.14	1.37 to 1.92	11.70	< 0.001	2	0.0 (0.768)
**60 months after TKA**							
Fixed effect	1.68	0.14	1.40 to 1.96	11.79	< 0.001		
Random effect	1.68	0.14	1.40 to 1.96	11.79	< 0.001	2	0.0 (0.512)

*CI, confidence interval; ES, effect size; I^2^, index of heterogeneity; n, number of included studies in analysis; SE, standard error; P, significance level; Q, test statistics.*

Two studies (4 *ES*s) measured MVS 14 days after surgery. Thus, MVS showed a most likely large harmful effect (*ES* = −1.77; 95% *CI* −2.13 to −1.41). The evidence was downgraded from high quality to moderate quality because of imprecision (sample size, <300) (Table 2). There was no significant heterogeneity (*Q* = 2.79; *p* = 0.455; *I*^2^ = 0%), which was why no further sub-analyses were conducted. In total, four studies (14 *ES*s) assessed the MVS 24–30 days post-surgery period where the most likely large harmful effect (*ES* = −1.47; 95% *CI* −2.01 to −0.94; *p* < 0.001) was observed. The evidence was downgraded from high quality to low quality because of imprecision (sample size < 300) and high heterogeneity (*I*^2^ = 81%) (Table 2). Because of the significantly large heterogeneity of the observed effect (*Q* = 58.16; *p* < 0.001; *I*^2^ = 81.1%), an additional sub-analysis was conducted, where the effects were determined of the pre-post-operative rehabilitation process and the BMI of patients, on the magnitude of knee extensors MVS. Thus, the OPGR protocol showed the most likely moderate harmful effect on MVS (*ES* = −1.04; 95% *CI* −1.42 to −0.66; *n* = 8; *p* < 0.001), while UC-rehabilitation showed the most likely extremely large harmful effect on MVS (*ES* = −4.27; 95% *CI* −6.51 to −2.03; *n* = 4; *p* < 0.001) following the post-surgery period. There was no study that investigated the effects of pre-surgery rehabilitation programs. Furthermore, patients who had a BMI of <30 kg/m^2^ showed a considerably smaller decrease in MVS (*ES* = −1.08; 95% *CI* −1.85 to −0.32; *n* = 6; *p* = 0.006) when compared to patients with a BMI of ≥30 kg/m^2^ (*ES* = −1.86; 95% *CI* −2.56 to −1.16; *n* = 5; *p* < 0.001).

#### Maximal Voluntary Isometric Contraction-Norm Recovery Following Total Knee Arthroplasty Surgery for Time Points of 1.5 Months and More

Given that the meta-regression analysis showed that time of evaluation was not a significant predictor of strength outcomes, three assessment periods following operation were summarized (1.5, 2, and 3 months). These data were pooled together and considered as a single time point. Thus, the summarized results of seven studies (20 *ES*s) showed a very likely small harmful effect on MVS for both the random- (*ES* = −0.38; 95% *CI* −0.60 to −0.17; *p* < 0.001) and fixed- (*ES* = −0.42; 95% *CI* −0.67 to −0.16; *p* < 0.001) effects models, respectively. The evidence was downgraded from high quality to low quality because of imprecision (sample size < 300) and moderate heterogeneity (*I*^2^ = 52%) (Table 2). Because of the significantly moderate heterogeneity of the observed effects (*Q* = 39.41, *p* = 0.004; *I*^2^ = 51.8%), an additional sub-analysis was performed. Comparing the effects of pre-rehabilitation (*ES* = −0.47; 95% *CI* −1.08 to 0.14; *n* = 2; *p* = 0.131), OPGR (*ES* = −0.37; 95% *CI* −0.69 to −0.06; *n* = 8; *p* = 0.02), and UC-rehabilitation (*ES* = −0.39; 95% *CI*−0.74 to −0.04; *n* = 10; *p* = 0.027), patients who were included in post-surgery rehabilitation programs seemed to have a similar decrease in strength, such as those from UC, but a slightly lesser magnitude of MVS decrease compared to pre-rehabilitation programs, respectively. Furthermore, patients with a BMI of ≥30 kg/m^2^ showed a tendency for greater decrease in MVS (*ES* = −0.43; 95% *CI* −0.65 to −0.21; *n* = 12; *p* = < 0.001) when compared to patients with a BMI of less than 30 kg/m^2^ (*ES* = −0.38; 95% *CI* −0.79 to 0.03; *n* = 8; *p* = 0.072).

In total, seven studies (14 *ES*s) aimed to investigate strength outcomes 6 months after the surgery. The summarized effect showed the most likely and possibly small beneficial effect on MVS for fixed (*ES* = 0.37; 95% *CI* 0.25 to 0.5; *p* < 0.001) and random effect (*ES* = 0.28; 95% *CI* −0.02 to 0.59; *p* = 0.063). The evidence was downgraded from high quality to moderate quality because of high heterogeneity (*I*^2^ = 79%) (Table 2). Because of significantly moderately large heterogeneity (*Q* = 61.96, *p* < 0.001; *I*^2^ = 79.1%), an additional sub-analysis was performed. Therefore, in the sub-analysis, we determined the effects of both the post-operative rehabilitation process and BMI of the patients on the magnitude of the knee extensors’ MVS. Thus, following the post-surgery period, the OPGR protocol showed a possibly trivial effect on MVS (*ES* = 0.15; 95% *CI* −0.06 to 0.35; *n* = 5; *p* = 0.156), while the UC-rehab showed a possibly small beneficial effect on MVS (*ES* = 0.31; 95% *CI* −0.13 to 0.76; *n* = 9; *p* = 0.168). There was no study that investigated the effects of pre-surgery rehabilitation programs. Furthermore, patients who had a BMI of <30 kg/m^2^ showed a considerably smaller increase in MVS (*ES* = 0.07; 95% *CI* −0.47 to 0.61; *n* = 6; *p* = 0.792) when compared to patients with a BMI of ≥30 kg/m^2^ (*ES* = 0.43; 95% *CI* 0.07 to 0.79; *n* = 8; *p* = 0.019). Since the between-study variance for patients with a BMI of ≥30 kg/m^2^ (*I*^2^ = 80.3%; *Q* = 35.45; *p* < 0.001) and <30 kg/m^2^ (*I*^2^ = 74.35%; *Q* = 19.49; *p* = 0.002) was large to moderate, no valid estimate of strength increase 6 months post-surgery could be confirmed.

Furthermore, in six studies (10 *ES*s), strength was assessed 1 year after the surgery. The fixed- and random-effects models showed the most likely moderate (*ES* = 0.9; 95% *CI* 0.72 to 1.24; *p* < 0.001) and very likely moderate beneficial effects on MVS (*ES* = 0.77; 95% *CI* 0.3 to 1.24; *p* = 0.001), respectively. The evidence was downgraded from high quality to moderate quality because of high heterogeneity (*I*^2^ = 85%) (Table 2). Although, high heterogeneity of the observed effects was seen (*Q* = 59.25; *p* < 0.001; *I*^2^ = 84.8%), the robustness of the conclusion concerning this time point could be drawn, because there were no considerable differences in the magnitude of the effects between the two applied models (i.e., fixed vs. random). In addition, we determined the effects of the post-operative rehabilitation process and BMI of the patients in the sub-analysis. Thus, the OPGR protocol showed a likely small beneficial effect on MVS (*ES* = 0.40; 95% *CI* 0.1 to 0.7; *n* = 4; *p* = 0.01), while the UC-rehabilitation showed a very likely moderate beneficial effect on MVS (*ES* = 0.93; 95% *CI* 0.29 to 1.57; *n* = 6; *p* = 0.004). There was no study that investigated the effects of pre-surgery rehabilitation programs. Furthermore, patients who had a BMI of <30 kg/m^2^ showed a considerably smaller increase in MVS (*ES* = 0.39; 95% *CI* 0.13 to 0.65; *n* = 5; *p* = 0.003) when compared to patients with a BMI of ≥30 kg/m^2^ (*ES* = 1.07; 95% *CI* 0.38 to 1.76; *n* = 5; *p* = 0.002). Although the between-study variance for patients with a BMI of ≥30 kg/m^2^ was statistically large (*Q* = 29.72; *p* < 0.001; *I*^2^ = 86.54%), the robustness of the conclusion between two BMI categories for this time point could be guaranteed, because there were no considerable discrepancies in the magnitude of the effects between the two applied models (i.e., fixed vs. random, *ES* = 1.32; 95% *CI* 1.08 to 1.55; *p* < 0.001 vs. *ES* = 1.07; 95% *CI* 0.38 to 1.76; *p* = 0.002).

Only one study (2 *ES*s) investigated the effects of TKA on MVS 2 years after surgery, suggesting a most likely large beneficial effect (*ES* = 1.64; 95% *CI* 1.37 to 1.92; *p* < 0.001). The heterogeneity of the observed effect was equal to 0%, suggesting robustness of the effect at this time point. Finally, long-term effects (i.e., 60 months post-surgery) were assessed in only one (2 *ES*s) study. The authors (61) showed a most likely large beneficial effect on MVS (*ES* = 1.68; 95% *CI* 1.4 to 1.96; *p* < 0.001; *I*^2^ = 0%). In both meta-analyses, the evidence was downgraded from high quality to moderate quality because of imprecision (sample size, <300) (Table 2).

### Maximal Voluntary Isometric Contraction-Norm of Patients With Total Knee Arthroplasty When Compared to Healthy Age-Matched Controls

Only two studies (2 *ES*s) included healthy age-matched control subjects for comparison. At pre-surgery, the patients had lower strength (*ES* = −3.41; 95% *CI* −7.06 to 0.25; *p* = 0.068). One month after surgery, the strength of the patients was most likely extremely lower than that of the controls (*ES* = −8.53; 95% *CI* −10.4 to −6.67; *n* = 1; *p* < 0.001). In the following months, the strength gradually increased to the level of the controls. However, although moderately lower strength was observed in the patients in the 12-month post-surgery period, the difference between the two groups was not significant (*ES* = −0.58; 95% *CI* −1.38 to 0.21; *n* = 1; *p* = 0.149; *I*^2^ = 0%) (Figure 3B).

### Meta-Regression Analysis

Table 4 shows the results of the univariate meta-regression for all included *ES*s considering the different time points of post-surgery evaluation for age of the patients, BMI, and sex ratio. The meta-regression conducted for all the included *ES*s showed that both the “post-surgery time of evaluation” (*Z* = 18.25; *R*^2^ = 0.38; *p* < 0.001) and the “age of the patients” (*Z* = 2.84; *R*^2^ = 0.07; *p* = 0.004) were significant predictors of MVS following surgery, suggesting that that more time passes by, the more the MVS recovers and that older patients were more likely to have greater benefits from post-surgery rehabilitation practice. Investigating the different post-surgery time point subcategories, none of the selected variables predicted the magnitude of MVS after 14 and 45 days, and up to 3 months after TKA. On the contrary, 21–30 days post-surgery, the age of the patients (*Z* = 2.43; *R*^2^ = 0.17; *p* = 0.015) and BMI (*Z* = −2.29; *R*^2^ = 0.152; *p* = 0.022), along with sex ratio (*Z* = −2.63; *R*^2^ = 0.21; *p* = 0.009), predicted the effect on MVS. Furthermore, the only predictor of the effect of TKA on MVS was the “age of the patients,” 6 months following surgery (*Z* = 2.43; *R*^2^ = 0.29; *p* = 0.015), while sex ratio was shown as a significant predictor of strength alterations 12 months post-surgery (*Z* = −2,15; *R*^2^ = 0.39; *p* = 0.031).

**TABLE 4:** Meta-regression for variables to predict muscle strength at different time points following total knee arthroplasty.

	Coefficient	Standard error	95% lower *CI*	95% upper *CI*	*Z* value	*R*square	*P* value
**Overall**							
Post-surgery time of evaluation	0.0458	0.0025	0.0409	0.0507	18.2461	**0.3787**	**<0.001**
Age of patients (years)	0.0893	0.0315	0.0276	0.1509	2.8381	0.0703	**0.0045**
BMI (kg/m^2^)	0.0986	0.0543	–0.0077	0.2050	1.8178	0.0331	0.0691
Sex ratio (males/females)	–0.4192	0.3100	–1.0268	0.1883	–1.3526	0.0182	0.1762
**21–30 days post-surgery**							
Post-surgery time of evaluation	0.8911	1.9702	–2.9705	4.7528	0.4523	0.0035	0.6511
Age of patients (years)	0.1413	0.0582	0.0273	0.2554	2.4283	**0.1693**	**0.0152**
BMI (kg/m^2^)	–0.2272	0.0994	–0.4220	–0.0323	–2.2851	**0.1520**	**0.0223**
Sex ratio (males/females)	–1.5759	0.5994	–2.7507	–0.4012	–2.6293	**0.2063**	**0.0086**
**45 days to 3 month post-surgery**							
Post-surgery time of evaluation	0.0039	0.0035	–0.0029	0.0108	1.1247	0.0382	0.2607
Age of patients (years)	0.0558	0.0293	–0.0015	0.1132	1.9086	0.1481	0.0563
BMI (kg/m^2^)	–0.0294	0.0391	–0.1061	0.0473	–0.7515	0,0252	0.4524
Sex ratio (males/females)	0.0388	0.2781	–0.5063	0.5839	0.1396	0.0009	0.8890
**6 months post-surgery**							
Age of patients (years)	0.0847	0.0349	0.0162	0.1532	2.4249	**0.2936**	**0.0153**
BMI (kg/m^2^)	0.0409	0.0976	–0.1504	0.2323	0.4193	0.0144	0.6750
Sex ratio (males/females)	–0.6286	0.3278	–1.2711	0.0141	–1.9171	0.2006	0.0552
**12 months post-surgery**							
Age of patients (years)	0.0957	0.0539	–0.0100	0.2015	1.7750	0.2999	0.0759
BMI (kg/m^2^)	0.1510	0.1274	–0.0987	0.4006	1.1853	0.1824	0.2359
Sex ratio (males/females)	–0.9445	0.4388	–1.8045	–0.0846	–2.1527	**0.3939**	**0.0313**

*BMI, body mass index (kg/m^2^). Bold value, statistically significant predictor of muscle strength following TKA.*

## Discussion

### Overall Findings of the Review and Meta-Analysis

The study presents a quantitative evaluation of quadriceps muscle strength recovery following TKA surgery using a meta-analysis approach based on data compiled and reviewed from previously published scientific research articles. We answered our primary hypotheses by showing that (i) the involved quadriceps MVS was recovered in a linear fashion and a time-dependent manner; (ii) within-subjects analysis showed that MVS recovered earlier than when it was compared to healthy age-matched controls; finally, (iii) patients’ age, sex, and BMI significantly moderated MVS outcomes following TKA. In detail, the present results show that in the early post-operative days, the quadriceps’ MVS markedly declines, after which it slowly recovers over time in a linear fashion. Thus, the greatest decline in MVS is observed 3 days after TKA, and in the following period, MVS is slowly recovered. However, compared to pre-operative values, it remains significantly impaired up to 3 months after TKA. Moreover, the sensitivity analysis using both the random- and fixed-effects models did not yield considerably different mean effects or *CI*s, suggesting that the results of the meta-analysis based on MViC data were robust. Furthermore, the meta-regression analysis showed that the time point of evaluation and patients’ age positively altered MVS, while on the contrary, the sex ratio (males/females) and BMI of the patients negatively moderated the quadriceps muscle MVS following TKA.

### Previous Findings on the Quadriceps Muscles’ Strength Recovery After Total Knee Arthroplasty

Previously, it was shown that the quadriceps muscles’ strength was the major determinant of functional recovery following TKA (2, 7, 17). Therefore, timely rehabilitation of MVS is necessary to attenuate functional performance decline and consequently improve the patients’ overall quality of life. Additionally, a considerable decrease in quadriceps strength was reported during the early post-operative period (e.g., 3 days to 3 months after surgery) (14, 48, 52, 56, 65), but the strength was gradually recovered over time; however, in some cases, the pre-operative levels were not reached, not even 6 months after surgery (21, 66). Our findings based on the meta-analytic approach showed similar trends, indicating that the quadriceps experienced substantial strength decreases in the first 3 months following TKA. Then, 12 months after TKA, the strength was recovered significantly when compared to pre-operative values, reached a plateau 2 years after surgery, and remained equal up to 60 months following the surgery.

### Factors That Influence the Quadriceps Muscles’ Strength Recovery After Total Knee Arthroplasty

The cause of muscle weakness (or decreased strength) after TKA has been associated with pain (71, 72), joint injury (73–75), and atherogenic muscle inhibition (76, 77) evoked by surgical trauma. However, the most commonly held belief as to why patients are weak early after surgery is assigned to pain, which causes failure to produce the maximal effort of volitional contraction of the quadriceps muscle. Pain is the most common symptom in patients suffering from knee OA, both before and one month following TKA (71, 78), and it is well-established that TKA can reliably reduce knee pain and improve knee function (78, 79) because of both physical therapy and pharmacological treatment pre- and post-surgery (14, 15, 80, 81). However, a substantial deficit in quadriceps strength still persists in the early months following TKA, regardless of the pain reduction present even during the early days after TKA (14, 56, 68). Thus, strength reduction should be primarily prescribed to other factors rather than pain during measurements (48).

Accordingly, it is well-known that both central and peripheral factors influence locomotion (82, 83) and, thus, MVS output (84–86). A recent study of Morita et al. (87) aimed to investigate the relationship between muscle weakness and activation of the cerebral cortex in the early period after unicompartmental knee arthroplasty, and it showed that muscle force was decreased by 50 and 37.5% following 1 and 2 weeks post-surgery, respectively. Strikingly, the active region of the sensorimotor leg area, assessed by functional near-infrared spectroscopy, narrowed as well (87), while the severity of the pain in knee joint assessed 2 weeks post-surgery did not significantly change, suggesting that the early post-operative muscle weakness was mostly influenced by the supraspinal level of the nervous system (87, 88).

One of the most investigated central factors related to MVS following TKA is represented by muscle voluntary activation (VA) level, whose failure represents a reduction in the maximal force output of the muscle due to inability to recruit all of the muscle’s motor units or failure to attain the maximal discharge rate from the recruited motor units (89, 90). The study of Mizner et al. (48) aimed to investigate the mechanism of early quadriceps MVS loss after TKA, and it showed that the combination of atrophy and failure of muscle VA explained approximately 85% of the quadriceps’ MVS loss. However, one month post-surgery, the relative contribution of VA was nearly twice as large compared to the relative contribution of muscle atrophy to the observed MVS decrease (48). Similar trends were also observed in two more recent studies (91, 92), suggesting that an early rehabilitation program should particularly focus on the recovery of VA deficit to attenuate the detrimental loss of muscle strength (93, 94).

Our findings, based on the summarized effects of post-rehabilitation practice, show that patients from the OPGR programs had a consistently lower decline in quadriceps MVS in the early periods (i.e., up to 3 months post-surgery) following TKA when compared to those from usual care. Similar results were observed when comparing pre-operative vs. outpatient professionally guided rehabilitation vs. usual care programs, showing that patients included in usual care rehabilitation programs experienced the greatest decline in MVS, followed by those in pre-operative and OPGR programs. Since OPGR includes an exercise that is more focused on quadriceps muscle strengthening, altering VA more than usual care, such as self-monitored physical practice, the present observed results recommend OPGR practice. Therefore, the findings of this study suggest that professionally guided post-rehabilitation and, possibly, pre-operative programs are more beneficial than outpatient self-monitored physical practice (i.e., usual care) for knee function improvement following TKA.

### Promising Interventions to Improve Quadriceps Muscles’ Strength Recovery After Total Knee Arthroplasty

There is mounting evidence that significant TKA rehabilitation progress can be made by starting it in the early hours after surgery (95, 96), incorporating neuromuscular electrical stimulation (NmES) and early intensive quadriceps strengthening in addition to the common physical therapy (20, 94, 97). Although NmES seems to be an applicable tool to attenuate quadriceps muscle MVS loss (20, 98), considerable differences among NmES studies in methodological considerations might confound the possibility to draw any firm conclusion (e.g., timing and duration of intervention, treatment volume, and intensity of NmES) (97). Given that only a small number of the included studies investigated NmES, direct comparisons of NmES with other post-rehabilitation programs were not conducted in this review (but actually were analyzed as a pooled intervention, i.e., OPGR). However, due to the already recognized potential of NmES treatment, the next paragraph will be devoted to NmES, with suggestions for improvement.

Due to the advent of technology, including neuroimaging and other brain activity measurement techniques, particularly functional magnetic resonance imaging and transcranial magnetic stimulation, the last two decades have been populated with studies investigating the neurological mechanisms of muscle-force regulation (86, 99). These neurophysiological studies support that NmES practice may affect the modulation of higher levels of the central nervous system (100, 101) [for a detailed review refer to Knutson et al. (102)]. Strikingly, when NmES is paired with actual movement execution, motor-related cortical potentials (MRCPs) showed to increase more than with NmES alone (103), suggesting that cortical excitability is improved by concurrent voluntary drive. Thus, combining the two practice methods simultaneously, patients could almost always activate not only the muscle but also the neural circuits controlling the motor action, adding a low-intensity actual movement execution exercise and/or cognitive training to NmES. Accordingly, the most recent studies suggest that cognitive training (i.e., motor imagery) alone (104), in addition to low-intensity strength training (105) or NmES (106), has a beneficial effect on strength increase in both the symptomatic and asymptomatic populations (107, 108). Thus, Jiang et al. (109) reported that at the end of a 12-week training of healthy elderly subjected to both high-intensity physical exercise and high mental effort (HME), elbow flexion strength was significantly increased compared to the control group, with no significant difference between the exercise-only and HME groups. The amount of increase in MRCPs in the HME group was significantly greater than in the exercise and control groups (109). Furthermore, combining the motor imagery practice with NmES in addition to common physical therapy was shown to be a beneficial practice to evoke re-learning of the motor task in patients who had stroke with unilateral neglect (106) compared to NmES only. Therefore, motor imagery may be a suitable additional tool for common rehabilitation practice in the early post-operative period, especially since patients who underwent TKA generally have impaired mobility and difficulty to participate in high-intensity exercise training programs (110, 111). This was further supported by recent studies (67, 112) that aimed to investigate the effects of motor imagery intervention on strength recovery following TKA. Paravlic et al. (67, 112) showed that adding motor imagery to routine physical therapy can attenuate strength decrease by 20.2% when compared to routine physical therapy alone. Moreover, later authors showed that failure of voluntary activation explained 47% quadriceps muscle strength loss, supporting the theory that motor imagery effects were governed by alterations at higher levels in the central nervous system.

To conclude, the holistic approach to rehabilitation of patients who underwent TKA, including both pre- and post-operative rehabilitation programs, should be administered to attenuate strength loss following TKA, while the post-rehabilitation practice may be improved by intensive multimodal approaches (53, 94).

### Recommended Treatments to Facilitate Quadriceps Muscle Strength Recovery After Total Knee Arthroplasty

Previous studies showed that typical characteristics of patients (i.e., sex and BMI) (29, 113) might influence the rehabilitation of patients who underwent TKA. Similarly, our findings suggest that age, sex, and BMI of patients influenced the outcomes of MVS. In the early days following TKA, BMI was negatively correlated with the observed effect, while, on the contrary, during a moderate-to long term period (i.e., 3–12 months after TKA), BMI was positively correlated with the effect. Moreover, the sub-analysis confirmed the meta-regression analysis findings, showing that in the early post-rehabilitation period, patients with a BMI of ≥30 kg/m^2^ had considerably greater MVS loss compared to those with a BMI of <30 kg/m^2^. However, 3, 6, and 12 months after TKA, they had greater MVS recovery rates. These results are similar to previous findings (114), which indicates that pre-surgery obese patients are more likely to reduce their BMI in the periods that follow compared to those with normal BMI (115). Therefore, weight management through diet manipulation should be included as part of a holistic rehabilitation process to attenuate the overall decrease in lower extremity function and quality of the patients’ life (116). An interesting finding from this study is that the age of the patients predicted the MVS outcomes 6 and 12 months following TKA (actually for the overall findings and early period after TKA), where positive relationships with the observed effects were seen. Thus, our results suggest that older patients experienced better recovery after TKA, which is in contrast to the physiological time course of recovery following muscle disuse when younger and older adults were compared (13). This might be ascribed to the greater attendance (e.g., persistence in self-monitored post-rehabilitation) of the assessed population. In general, inter-individual differences in sociopsychological characteristics such as marital status and low social participation have been shown to play an important role in intervention attendance (117). Additionally, it could be that the decline in muscle strength is smaller in the elderly because of slower physiological processes when compared to younger patients (13). Therefore, younger patients who underwent TKA should be encouraged to meticulously follow and complete all the phases of pre- and post-rehabilitation programs in order to achieve the most optimal results, even if they already feel strong enough in any phase. Another patient characteristic that was shown to be a moderator of post-TKA outcomes is patient’s sex. In previous studies, it was found that women have less optimal functional outcomes after TKA than men (36, 37). Our meta-regression showed that sex-ratio considerably influenced the effects of TKA on MVS 21–30 days and 12 months post-surgery. Therefore, future studies should conduct a sex-specific analysis, since this characteristic might have a significant influence on rehabilitation outcomes following TKA.

## Study Limitations

This study lacks a randomized control group comparison. Randomized control trials (RCTs) are certainly desirable to improve our understanding of the effects of TKA on strength; however, this kind of study design is relatively rare in surgery (118). Second, the patients from the included studies differed in terms of surgery type (i.e., incision location, prosthesis used, patella resurfacing or not), not allowing clear distinction among the surgery approaches, and, thus, no particular influence on strength outcome could be drawn. Third, investigation of possible reasons why a patient characteristic such as age, sex, and BMI represents a significant moderating factor of MVS recovery following TKA is warranted in future studies.

## Conclusion

From the reviewed literature on TKA strength recovery, we observed a considerable decrease in the involved quadriceps muscle strength following surgery, which lasted for several months post-surgery. Furthermore, age sex, and BMI of the patients might significantly influence the outcomes of TKA. Therefore, our data suggest that strategies specifically targeting the strengthening of knee extensor muscles and preserve the neural circuits of motor control, along with nutritional management, may need to be incorporated in early rehabilitation to improve the outcomes following TKA.

## Clinical Messages

• Patients who received outpatient professionally guided rehabilitation practice had a consistently lower decline in knee extensor strength in the early periods (i.e., up to 3 months post-surgery) following TKA when compared to patients who received usual care.
• Age, BMI, and sex of patients are noted as significant moderators of strength outcomes following TKA. Therefore, future studies should control the effects of TKA and corresponding rehabilitation outcomes for age, BMI, and sex.
• Strategies that preserve the neural circuits of motor control along with proper nutritional interventions following TKA are highly recommended.

## Data Availability Statement

The original contributions presented in the study are included in the article/Supplementary Material, further inquiries can be directed to the corresponding author.

## Author Contributions

AP had given substantial contributions to the conception of the manuscript, analysis, and data interpretation. KD and CM contributed to acquisition and interpretation of the data. All authors have participated in drafting the manuscript, while KD and CM revised it critically. All authors contributed to the article and approved the submitted version.

## Conflict of Interest

The authors declare that the research was conducted in the absence of any commercial or financial relationships that could be construed as a potential conflict of interest.

## Publisher’s Note

All claims expressed in this article are solely those of the authors and do not necessarily represent those of their affiliated organizations, or those of the publisher, the editors and the reviewers. Any product that may be evaluated in this article, or claim that may be made by its manufacturer, is not guaranteed or endorsed by the publisher.
