# Supplementary material for: The Time Course of Quadriceps Strength Recovery After Total Knee Arthroplasty Is Influenced by Body Mass Index, Sex, and Age of Patients: Systematic Review and Meta-Analysis
Source: Front Med (Lausanne). 2022 May 25;9:865412. doi: 10.3389/fmed.2022.865412 (PMC9174520; doi:10.3389/fmed.2022.865412)
Supplement: Supplementary file 1 [file Data_Sheet_1.pdf]

Searching strategy for different databases:

General key words used:

total knee arthroplasty, knee replacement surgery, functional performance, functional impairment, quadriceps, knee extensors muscles, muscle strength, rehabilitation, BMI.

PubMed

Total numbers of the articles found: 754

Search: **((total knee arthroplasty) OR (knee replacement surgery)) AND (muscle strength)**
("arthroplasty, replacement, knee"[MeSH Terms] OR ("arthroplasty"[All Fields] AND "replacement"[All Fields] AND "knee"[All Fields]) OR "knee replacement arthroplasty"[All Fields] OR ("total"[All Fields] AND "knee"[All Fields] AND "arthroplasty"[All Fields]) OR "total knee arthroplasty"[All Fields] OR (("arthroplasty, replacement, knee"[MeSH Terms] OR ("arthroplasty"[All Fields] AND "replacement"[All Fields] AND "knee"[All Fields]) OR "knee replacement arthroplasty"[All Fields] OR ("knee"[All Fields] AND "replacement"[All Fields]) OR "knee replacement"[All Fields]) AND ("surgery"[MeSH Subheading] OR "surgery"[All Fields] OR "surgical procedures, operative"[MeSH Terms] OR ("surgical"[All Fields] AND "procedures"[All Fields] AND "operative"[All Fields]) OR "operative surgical procedures"[All Fields] OR "general surgery"[MeSH Terms] OR ("general"[All Fields] AND "surgery"[All Fields]) OR "general surgery"[All Fields] OR "surgery s"[All Fields] OR "surgerys"[All Fields] OR "surgeries"[All Fields]))) AND ("muscle strength"[MeSH Terms] OR ("muscle"[All Fields] AND "strength"[All Fields]) OR "muscle strength"[All Fields])
Translations
total knee arthroplasty: "arthroplasty, replacement, knee"[MeSH Terms] OR ("arthroplasty"[All Fields] AND "replacement"[All Fields] AND "knee"[All Fields]) OR "knee replacement arthroplasty"[All Fields] OR ("total"[All Fields] AND "knee"[All Fields] AND "arthroplasty"[All Fields]) OR "total knee arthroplasty"[All Fields]
knee replacement: "arthroplasty, replacement, knee"[MeSH Terms] OR ("arthroplasty"[All Fields] AND "replacement"[All Fields] AND "knee"[All Fields]) OR "knee replacement arthroplasty"[All Fields] OR ("knee"[All Fields] AND "replacement"[All Fields]) OR "knee replacement"[All Fields]
surgery: "surgery"[Subheading] OR "surgery"[All Fields] OR "surgical procedures, operative"[MeSH Terms] OR ("surgical"[All Fields] AND "procedures"[All Fields] AND "operative"[All Fields]) OR "operative surgical procedures"[All Fields] OR "general surgery"[MeSH Terms] OR ("general"[All Fields] AND "surgery"[All Fields]) OR "general surgery"[All Fields] OR "surgery's"[All Fields] OR "surgerys"[All Fields] OR "surgeries"[All Fields]
muscle strength: "muscle strength"[MeSH Terms] OR ("muscle"[All Fields] AND "strength"[All Fields]) OR "muscle strength"[All Fields]

Cochraine

https://www.cochranelibrary.com/advanced-search

3 Trials matching **total knee arthroplasty, knee replacement surgery, functional performance, quadriceps, knee extensor muscles, muscle strength, rehabilitation in Title Abstract Keyword - (Word variations have been searched)**

Google Scholar
Total numbers of the articles found: 2930
Key words: total knee arthroplasty, total knee replacement surgery, muscle strength, torque, force, knee extensors, functional performance, quadriceps, rehabilitation

Other databases together: 325 articles
