# Supplementary material for: The Time Course of Quadriceps Strength Recovery After Total Knee Arthroplasty Is Influenced by Body Mass Index, Sex, and Age of Patients: Systematic Review and Meta-Analysis
Source: Front Med (Lausanne). 2022 May 25;9:865412. doi: 10.3389/fmed.2022.865412 (PMC9174520; doi:10.3389/fmed.2022.865412)
Supplement: Supplementary file 2 [file Table_1.pdf]

Table 1 Systematic review showing the characteristics of the included studies selected for meta-analysis (n = 17) with their relevant outcomes

Study	Outcome measures	Population					Time post	Results ↑↓→	Additional Comments
	Outcome measure; Knee angle	Age (years) [mean ± SD]	BMI (kg/m^2)	Sex (ratio)	Arthroplasty	Sample size	Period post-operation		
**Bade and Stevens-Lapsley** (52)	Knee extension (MViC) normalized by BW in kg<br><br>60°	HIRT<br>65.3 ± 11.5	29.7 ± 4.6	NR	Primary unilateral TKA<br><br>**Surgical approach**: NR.	B 8; A 8	3.5 weeks	-23.1	Group differentiated regarding post-operative rehabilitation (i.e., high intensity and low-intensity rehabilitation program).
						=; =	6.5 weeks	-15.4	
		LIRT<br>65 ± 11.5	30.9 ± 3.4	NR		=; =	3 months	7.7	
						=; =	6 months	30.8	
						=; =	12 months	30.8	
						B 8; A 8	3.5 weeks	-50.0	
						=; =	6.5 weeks	-25.0	
						=; =	3 months	-16.7	
						=; =	6 months	0.0	
						=; =	12 months	16.7	
**Collados-Maestre1 et al.** (61)	Knee extensors strength (MViC) normalized by BW in kg<br><br>NR	SR 71.9 ± 6.3	30.6 ± 3.9	M/F (37/81)	Primary unilateral TKA<br><br>**Surgical approach:** All patients in both groups received patellar resurfacing. Standard antibiotic, antithrombotic prophylaxis.	B 118; A 118	6 months	37.5 ↑	Usual postoperative rehabilitation was given to all patients.
						=; =	12 months	81.3 ↑	
						=; =	2 years	100.0 ↑	
				M/F (35/84)		=; =	5 years	87.5 ↑	
		MR 70.6 ± 6.5	31.3 ± 3.4			B 119; A 119	6 months	28.6 ↑	
						=; =	12 months	85.7 ↑	
							2 years	92.9 ↑	

						=; =<br>=; =	5 years	85.7 ↑	
**Holm et al.** (65)	Knee extensors strength (MViC) normalized by BW in kg<br><br>60°	UC 66.0 ± 7.0	28.1 ± 4.2	M/F (11/13)	Primary unilateral TKA<br>**Surgical approach:** Medial parapatellar approach (including resurfacing of the patella)	B 24; A 24	3 days	- 80 ↓	All patients followed a fast-track program for TKA. It included preoperative multidisciplinary education, a specialized ward for patients receiving arthroplasty only, well-defined optimized multimodal pain treatment, postoperative rehabilitation that included early ambulation, and early oral nutrition.
**McKay et al.** (56)	Knee extensors strength (MViC) normalized by kg of body weight<br><br>75°	RT 63.5 ± 4.93<br><br>CON 60.58 ± 8.05	35.03 ± 6.13<br><br>35.03 ± 6.13	M/F (5/5)<br><br>M/F (4/8)	Primary unilateral TKA<br>**Surgical approach:** NR	B 10; A 10<br>=; =<br>B 12; A 12<br>=; =	6 weeks<br>3 months<br>6 weeks<br>3 months	-37,5<br>-19,8<br>-32,1<br>-11,9	TKA primary because of OA. No symptoms of OA in the contralateral knee. Group differentiated regarding pre-operative rehabilitation (i.e., RT or CON-placebo RT [strength training for upper body]).
**Mizner et al.** (48)	Knee extensors strength (MViC) normalized by BMI<br><br>75°	62.0 ± 8.0	31.0 ± 5.0	M/F (12/8)	Primary unilateral TKA<br>**Surgical approach:** A tricompartmental, cemented TKA with a medial parapatellar surgical approach.	B 20; A 20<br>=; =	27 days		TKA was performed primary because of OA. Subjects who had substantial impairment in any of the other lower-extremity joints were excluded.<br>Postoperatively, all subjects underwent standardized inpatient and home-therapy protocols before testing and were functioning clinically.
**Mizner et al.** (17)	Knee extensors strength (MViC) normalized by BMI<br><br>75°	OPGR 63.8 ± 8	29.4 ± 4.2	M/F (25/15)	Primary unilateral TKA<br>**Surgical approach:** A tricompartmental, cemented TKA with a medial parapatellar surgical approach.	B 40; A 40	12 months	8.7	TKA was performed primary because of OA. Following the TKA, subjects underwent 3 days of inpatient physical therapy, followed by 2 to 3 weeks of home physical therapy visits. At approximately 4 weeks after surgery, subjects started 6 weeks (2 to 3 times per week; mean, 17 visits) of outpatient professionally guided rehabilitation.

**Mizner et al.** (7)	Knee extensors strength (MViC) normalized by BMI<br><br>75°	OPGR 64.0 ± 9.0	31.4 ± 3.7	M/F (22/18)	Primary unilateral TKA<br>**Surgical approach:** A tricompartmental, cemented TKA with a medial parapatellar surgical approach.	B 40; A 40<br>=; =<br>=; =<br>=; =	1 month<br>2 months<br>3 months<br>6 months	-61.1 ↓<br>-38.9 ↓<br>-16.7 ↓<br>0.0 →	=
**Mizner et al.** (68)	Knee extensors strength (MViC) normalized by BMI<br><br>75°	OPGR 65.0 ± 9.0	30.8 ± 4.5	M/F (52/48)	Primary unilateral TKA<br>**Surgical approach:** A tricompartmental, cemented TKA with a medial parapatellar surgical approach.	B 100; A 100<br>=; =	1 month<br>12 months	-47.7 ↓<br>4.7 ↑	=
**Nutton et al.** (57)	Knee extensors strength (MViC) normalized by kg of body weight<br><br>90°	SMP 71.0 ± 7.0<br><br>MV 74.0. ± 8.0	28.9 ± 2.6<br><br>31.2 ± 4.3	M/F (7/5)<br><br>M/F (7/4)	Primary unilateral TKA<br>**Surgical approach:** SMP or MV approach without patellar resurfacing	B 12; A 12<br>=; =<br>=; =<br>B 11; A 11<br>=; =<br>=; =	6 weeks<br>3 months<br>6 months<br>6 weeks<br>3 months<br>6 months	-17.3 ↓<br>-7.7 ↓<br>17.3 ↑<br>13.3 ↑<br>15.6 ↑<br>42.2 ↑	TKA was performed primary because of OA. Group differentiated regarding surgical approach (i.e., SMP or MV). The postoperative care for both of the patient groups was identical, using a standard care plan which emphasizes early weight-bearing and knee flexion exercises.
**Pua et al.** (49)	Knee extensors strength (MViC), normalized by BW in kg<br><br>75°	UC 67.0 ± 8.0	26.0 ± 4.8	M/F (24/61)	Primary unilateral TKA<br>**Surgical approach:** NR	B 85; A 85<br>=; =	14 days<br>3 months	-53.6 ↓<br>-8.0 ↓	TKA was performed primary because of OA. From postoperative days 2 to 4, patients underwent additional quadriceps muscle NmES or active quadriceps exercises (control treatment). Because there were no significant differences between treatment conditions in all outcomes, results from this study were presented based on the entire sample.
**Smith et al.** (22)	Knee extensors strength (MViC)	UC 62.7 ± 6.8	31.0 ± 4.1	M/F (2/11)	Primary unilateral TKA<br>**Surgical approach:** All procedures were performed using a medial parapatellar	B 13; A 13<br>=; =	6 months<br>12 months	18.1 ↑<br>29.4 ↑	TKA was performed primary because of OA. Additional inclusion criteria for the TKA-group subjects included diagnosis of OA in both knees, which was

	normalized by BMI<br><br>45°				arthrotomy, while implants consisted of cemented or cementless cruciate retaining femoral components, cemented modular titanium tibial components and either a CR or anterior stabilized bearing.				confirmed pre-operatively with radiographs.
**Stevens et al.** (69)	Knee extensors strength (MViC) Normalized by Nm/BMI<br><br>75°	UC 63.0 ± 8.8	NR		Primary unilateral TKA<br>**Surgical approach:** Medial parapatellar approach and cemented insertion of the patellar. tibia1 and femoral components were used	B 28; A 28	26 days	-60,0 ↓	TKA was performed primary because of OA. Those patients with symptomatic contralateral knee OA were excluded.<br>The post-operative regimen included inpatient hospitalization for four days followed by 2 weeks (six visits) of home physical therapy.
**Stevens-Lapsley et al.** (66)	Knee extensors strength (MViC) normalized by BW in kg<br><br>60°	OPGR 64.3 ± 9.2	29.8 ± 4.3	M/F (17/13)	Primary unilateral TKA<br>**Surgical approach:** Medial parapatellar approach and PCL-sparing, cemented, modular fixed-bearing components were used.	B 30; A 30<br>=; =<br>=; =	1 month<br>3 months<br>6 months	-55.2 ↓<br>-19.5 ↓<br>-9.3 ↓	Patients with symptomatic contralateral knee OA were excluded.<br>All postoperative rehabilitation was standardized using progressive exercises focused on quadriceps and hamstrings muscle strength.<br>Inpatient rehabilitation (3–4 days) was followed by 2 weeks of home physical therapy (six to seven visits), after which patients proceeded to outpatient physical therapy for an average of 10 additional visits.
**Stevens-Lapsley et al.** (20)	Knee extensors strength (MViC) normalized by BW in kg<br><br>60°	OPGR (NmES)<br>66.2 ± 9.1<br><br>UC 64.8 ± 7.7	27.1 ± 4.9<br><br>31.2 ± 4.2	M/F (15/20)<br><br>M/F (15/16)	Primary unilateral TKA<br>**Surgical approach:** All patients underwent a tricompartmental, cemented TKA with a medial parapatellar surgical approach.<br>=	B 35; A 31<br>=; A 31<br>=; A 30<br>=; A 31<br>=; A 30<br>B 31; A 28	3,5 weeks<br>6,5 weeks<br>13 weeks<br>26 weeks<br>52 weeks<br>3,5 weeks	-30.1<br>-9.8<br>6.8<br>13.5<br>24.8<br>-50.0	Patients with symptomatic contralateral knee OA were excluded. Standard inpatient rehabilitation began on postoperative day 1 and continued twice daily for 3 days. All patients were provided with the same standard rehabilitation protocol for TKA, consisting of a defined set of core exercises. Following hospital

						=; A 28	6,5 weeks	-21.2	discharge, participants received 6 treatments at home over 2 weeks and then received 10 to 12 outpatient physical therapy visits.
						=; A 29	13 weeks	-9.1	
						=; A 27	26 weeks	5.3	
						=; A 25	52 weeks	13.6	
**Wada et al.** (60)	Knee extensors strength (MViC) normalized by BW in kg<br>75°	UC WD 70.8 ± 7.2	27.6 ± 2.2	M/F (10/10)	Primary unilateral TKA<br>**Surgical approach:** NR	B 20; A 20	6 months	11.7 ↑	TKA was performed primary because of OA. Patients in both groups underwent the same rehabilitation program, which consisted of a standard 5-day inpatient and 12-week outpatient rehabilitation program.
						=; =	12 months	20.4 ↑	
		UC D 71.8 ± 7.3	76.7 ± 3.9	M/F (10/10)		B 20; A 20	6 months	3.1 ↑	
						=; =	12 months	11.3 ↑	
**Yoshida et al.** (51)	Knee extensors strength (MViC) normalized by BW in kg<br>90°	sNmES 71.6 ± 7	25.4 ± 2.2	M/F (18/4)	Primary unilateral TKA<br>**Surgical approach:** Medial parapatellar approach with cemented tricompartmental prosthesis with fixed bearing (including resurfacing of the patella).	B 22; A 22	14 days	-58.6 ↓	TKA was performed primary because of OA. All patients received usual physical therapy, while two groups have the additional sensory level intensity of maximal intensity NmES.
							1 month	-17.2 ↓	
		mNmES 75.9 ± 4.7	24.6 ± 2.9	M/F (18/4)		=; =	2 weeks	-55.2 ↓	
							1 month	-10.3 ↓	
		CON	25.8 ± 3.3	M/F (20/2)		=; =	2 weeks	-56.7 ↓	
							1 month	-36.7 ↓	
**Paravlic et al.** (67)	Knee extensors strength (MViC) normalized by BW in kg<br>60°	MI 62.7 ± 5.2	30.5 ± 4.0	M/F (7/6)	Primary unilateral TKA<br>**Surgical approach:** Medial parapatellar approach with cemented tricompartmental prosthesis with fixed bearing	B 13; A 13	1 month	-42.3↓	TKA was performed primary because of OA. All patients received usual physical therapy, while MI group had the additional motor imagery intervention
		CON 58.9 ± 5.2	30.1 ± 1.8	M/F (7/6)		B 13; A 13	1 month	-62.5↓	

A after surgery, B before surgery, BMI body mass index, CON control, SD standard deviation, MVC Maximum Voluntary Contraction, MViC Maximal Voluntary isometric Contraction, NR not reported, TKA total knee arthroplasty, M male, F female, RT resistance training, PRT Progressive resistance training, ST standard training, UC usual care, OA osteoarthritis, BW body weight, SR TKA single radius TKA, SMP standard medial parapatellar TKA, MIS minimally invasive TKA, OPGR outpatient professionally guided rehabilitation, NmES neuromuscular electro stimulation, sNmES sensory-level intensity NmES, mNmES maximal intensity NmES, AQ aquatic therapy group, MSV mini subvastus surgery approach, PSRP pre-surgery rehabilitation program, MR TKA multi-radius TKA, MV midvastus surgery approach, PE patellar eversion technique, WPE without patellar eversion technique, KLS Kellgren– Lawrence Scale, D patients with diabetes, WD patients without diabetes, HIRT high intensity rehabilitation therapy LIRT low intensity rehabilitation therapy, ROM range of motion

↑ Increase in strength, ↓ decrease in strength, → strength remained the same
